# Sleep in Young Children With Autism, ADHD and Combined Presentations

**DOI:** 10.1002/aur.70326

**Published:** 2026-07-25

**Authors:** Eva‐Maria Kurz, Sven Bölte, Terje Falck‐Ytter

**Affiliations:** ^1^ Development and Neurodiversity Lab, Department of Psychology Uppsala University Uppsala Sweden; ^2^ Center of Neurodevelopmental Disorders (KIND), Division of Neuropsychiatry, Department of Women's and Children's Health Karolinska Institute Stockholm Sweden; ^3^ Child and Adolescent Psychiatry Stockholm Health Care Services Stockholm Sweden; ^4^ Curtin Autism Research Group, Curtin School of Allied Health Curtin University Perth Western Australia Australia

**Keywords:** ADHD, AuDHD, autism, sleep, transdiagnostic

## Abstract

Autism and attention deficit hyperactivity disorder (ADHD) are often accompanied by sleep problems. Longitudinal studies hint towards a greater proportion of persisting sleep problems across early development in neurodivergent compared to neurotypical individuals. Within the context of a prospective infant sibling study of autism and ADHD (*n* = 209), we compared early caregiver‐rated sleep trajectories of individuals diagnosed with autism, ADHD or cooccurring autism and ADHD (AuDHD) at 36 months with two groups that did not meet diagnostic criteria—one group of individuals with and one without elevated likelihood of autism and/or ADHD. Sleep behaviors assessed at 10 and 14 months (i.e., number of night awakenings and settle durations) did not differ between individuals with and without a diagnosis of autism and/or ADHD. At 24 and 36 months, individuals diagnosed with autism and/or ADHD took longer to fall asleep, had less sufficient sleep, more parasomnia‐related behaviors and had greater overall sleep disturbance than those without a diagnosis, suggesting that their sleep problems are more circumscribed than often reported for older age ranges. In autism specifically, bedtime resistance improved from 24 to 36 months. While additional studies incorporating objective sleep measures are needed, the current results indicate that sleep involvement in neurodevelopmental conditions may mainly reflect transdiagnostic rather than diagnosis‐specific processes in early childhood.

## Summary

Both autism and attention deficit hyperactivity disorder (ADHD) are often accompanied by sleep problems although findings from early childhood are mixed and little is known about individuals with cooccurring autism and ADHD (AuDHD). During toddlerhood subtle differences regarding a few specific caregiver‐rated sleep behaviors emerged between individuals with a diagnosis of autism and/or ADHD compared to those without a diagnosis. Overall, these findings suggest that emerging sleep problems may reflect processes shared across autism and ADHD rather than patterns that are unique to either diagnosis.

## 1 Introduction

Autism and attention deficit hyperactivity disorder (ADHD) are neurodevelopmental conditions that are often accompanied by altered sleep, including longer sleep onset latencies, more night awakenings, and reduced sleep efficiency and quality in both individuals with autism and ADHD as compared to neurotypical individuals (Cortese et al. 2009; Díaz‐Román et al. 2018; Elrod and Hood 2015; Marten et al. 2023). Rates for an accompanying sleep problem range from 25 up to 80% in children with autism or ADHD (Bruni et al. 2025; Singh and Zimmerman 2015). Accordingly, the prevalence for any diagnosed sleep disorder such as insomnia, circadian rhythm disorder, sleep‐related breathing or movement disorder is elevated in autism (9%–17%) and ADHD (7.5%) (Ahlberg et al. 2023; Lai et al. 2019).

In the general population, around 15%–30% of caregivers rate at least one aspect of their infant's sleep behavior as problematic (Bayer et al. 2007; Williamson et al. 2019). While these sleep problems tend to decrease during childhood in the general population (Valla et al. 2022; Williamson et al. 2019), they appear to worsen in autistic children (Verhoeff et al. 2018). Although it has been shown that infants later diagnosed with autism have greater difficulties falling asleep at 6 and 12 months than infants without a diagnosis (MacDuffie et al. 2020), another study found differences regarding nighttime sleep between those with and without a later diagnosis of autism only at specific time points during infancy (i.e., at 14 but not at 5 and 10 months) (Begum‐Ali et al. 2023). Two longitudinal studies covering an age range from 1.5 to 9 years (Verhoeff et al. 2018) and 2–17 years (Sadikova et al. 2023), respectively, found that baseline sleep problems did not affect later autism symptom/trait expression but baseline autism phenotypic expression was associated with more sleep problems later on (Sadikova et al. 2023; Verhoeff et al. 2018).

A similarly, relatively stable prevalence of sleep problems from birth to 7 years was found in individuals later diagnosed with ADHD (Williams and Sciberras 2016). Furthermore, children with ADHD with persistent sleep problems across a one‐year period (10% of the sample) showed greater ADHD symptom/trait expression and co‐occurring internalizing and externalizing behavior challenges than those with transient or without sleep problems (Lycett et al. 2014, 2016). Several longitudinal studies found an association between early short sleep duration and later ADHD (Morales‐Muñoz et al. 2023; Scott et al. 2013; Tso et al. 2019). Contrarily, Carpena et al. (2020), did not find an association between early sleep duration and later ADHD but between increased nightmares, difficulties going to sleep as well as restless sleep with ADHD (Carpena et al. 2020). In another study, consistent sleep difficulties between 2 and 6 years were associated with an increased likelihood of ADHD at 11 years (Carpena et al. 2022).

Autism and ADHD frequently co‐occur (Rong et al. 2021). Both conditions are highly heritable (Faraone and Larsson 2019; Tick et al. 2016), and individuals with cooccurring autism and ADHD (AuDHD) have been found to be double‐loaded with genetic predispositions for both conditions (Mattheisen et al. 2022). Given the high prevalence of sleep problems in both conditions (Singh and Zimmerman 2015), it is reasonable to hypothesize that the likelihood for sleep problems is unproportionally increased in individuals with AuDHD. A comparison of children (5–13 years) with AuDHD versus ADHD revealed comparable sleep outcomes in both groups and evidence that cooccurring externalizing (oppositional defiant and/or conduct disorder) and internalizing (anxiety and/or mood disorders) disorders are associated with sleep problems in both groups (Thomas et al. 2018). Similarly, Reynolds et al. (2017) found no association between ADHD or autism phenotypic expression and sleep problems, but rather an influence of internalizing challenges on sleep in a sample of school‐aged children with AuDHD. Using a wearable device for assessing sleep and circadian parameters, a recent study found overall comparable sleep patterns in autistic children and children with AuDHD, except that children with AuDHD were more exposed to outdoor light, showed more time in movement, and had a later sleep midpoint (chronotype) than the autistic group (Martinez‐Cayuelas et al. 2024). Overall, there were more differences between the autism and comparison group than between the AuDHD and the typically developing group (Martinez‐Cayuelas et al. 2024). Altogether, evidence is scarce and existing studies focused on cross‐sectional comparisons of school‐aged children. Hence, how sleep is related to coexisting autism and ADHD in early childhood is largely unknown. The general high overlap of cooccurring sleep problems in neurodivergent individuals suggests that sleep could be a potential transdiagnostic mechanism (Astle et al. 2022) and target for transdiagnostic intervention approaches (Rigney et al. 2018).

The aim of the current study was to characterize early caregiver‐reported sleep in a sample of individuals from families with a history of autism and/or ADHD that were later diagnosed with either autism, ADHD, AuDHD, or did not meet diagnostic criteria. Sleep behaviors during infancy (at age 10 and 14 months) were assessed with a different questionnaire than during toddlerhood (at age 24 and 36 months) due to the use of questionnaires suitable for the respective ages. Given the paucity of research on longitudinal sleep trajectories in AuDHD and inconclusive findings in ADHD and autism, we did not have a hypothesis regarding specific differences between clinical groups or their trajectories across early childhood. Still, based on findings that sleep problems tend to improve across development in typically developing individuals, we expected a group of individuals without a diagnosis of autism and/or ADHD to show a greater reduction in indicators of problematic sleep across the investigated time points than the clinical groups. Exploratively, we investigated whether changes in sleep behaviors within the infant and toddler period were associated with autism and ADHD traits, as well as internalizing and externalizing behavioral challenges. Research questions and the analytic approach were pre‐registered at https://osf.io/v8tdk/.

## 2 Methods

### 2.1 Participants

Participants took part in the Early Autism Sweden Project (EASE; e.g., Hardiansyah et al. 2023; Nyström et al. 2019), a longitudinal study including infants at elevated familial likelihood (EL) of autism and/or ADHD as well as infants with low likelihood (LL) of autism and/or ADHD. Participants were recruited in the greater urban Stockholm area, Sweden, where potential EL families were recruited via direct outreach and adverts in pediatric clinics and potential LL families via the Swedish Population Registry. Participants in the EL group had to have at least a full sibling or parent with autism and/or ADHD. Five participants were included in the EL group despite having only a half‐sibling with autism and/or ADHD. Exclusion criteria specific to the LL group were the presence of autism, ADHD, or a developmental language disorder in a first or second degree relative as well as autism‐related concerns about the infant by the caregiver. General exclusion criteria were prematurity (born pre 36 weeks gestational age), the known presence of an autism‐related genetic syndrome, uncorrected vision or hearing impairments, epilepsy, developmental or medical conditions that affect brain development, and none of the caregivers speaking the testing language to the child at home. Written informed consent was obtained from all caregivers. The Stockholm Regional Ethics Committee approved the study (2010/2085‐31/3).

For a total of 240 participants diagnostic information was available at 36 months. Of those, two were excluded due to uncorrected hearing impairments and a medical condition with probable impact on cognitive functioning, respectively. Two participants were excluded because they were recruited as LL but received a diagnosis at 36 months. Another 27 participants were excluded because they did not provide any information on sleep behaviors at 10, 14, 24, or 36 months. The remaining 209 participants had sleep data available for at least one timepoint and were grouped into one of five groups based on the outcome of the diagnostic session at 36 months (see also diagnostic assessment): elevated likelihood with ADHD diagnosis (EL‐ADHD, *n* = 22, 36.4% female), elevated likelihood with autism diagnosis (EL‐autism, *n* = 29, 41.4% female), elevated likelihood with cooccurring autism and ADHD diagnosis (EL‐AuDHD, *n* = 8, 25% female), elevated likelihood without a diagnosis (EL‐none, *n* = 109, 50.5% female), and low likelihood without a diagnosis (LL‐none, *n* = 41, 53.7% female, see also Table 1).

**TABLE 1:** Demographic information.

	EL‐ADHD			EL‐Autism			EL‐AuDHD			EL‐none			LL‐none			*p* ^a^
	*n*	*M*	SEM	*n*	*M*	SEM	*n*	*M*	SEM	*n*	*M*	SEM	*n*	*M*	SEM	
Age (days)																
10 months	14	305	5.05	15	308	3.74	7	309	7.30	72	309	1.75	24	302	2.30	0.244
14 months	14	433	8.81	16	424	3.35	8	431	3.61	80	431	2.06	24	433	4.91	0.587
24 months	18	741	10.02	19	748	9.04	8	738	13.24	79	745	3.51	28	749	9.15	0.868
36 months	19	1145	16.61	28	1148	16.62	8	1143	27.64	101	1149	8.97	37	1130	11.30	0.995
Income^b^	19	4.63	0.36	27	4.89	0.30	7	5.57	0.69	102	5.13	0.17	39	5.13	0.26	0.590
Maternal education^c^	22	3	0.22	26	3.15	0.22	8	3.38	0.18	106	3.44	0.08	39	3.67	0.12	0.032
Paternal education^c^	19	2.95	0.18	24	2.79	0.18	8	2.88	0.35	96	3.16	0.10	33	3.21	0.18	0.265
MSEL^d^	22	93	3.69	27	86	4.58	8	78	5.31	109	105	1.46	39	119	2.21	< 0.001
ADOS‐2 CS^e^	22	4.32	0.46	29	6.62	0.36	8	6.88	0.52	109	3.08	0.17	39	2.44	0.25	< 0.001
ADHD rating scale	17	8.77	1.36	21	5.52	0.85	8	9.75	1.87	80	2.59	0.32	24	1.21	0.48	< 0.001
CBCL—Externalizing	21	16.24	2.14	29	18.76	2.02	8	18.00	3.09	107	10.02	0.77	40	6.55	1.00	< 0.001
CBCL—Internalizing	21	8.24	1.26	29	14.10	2.03	8	11.00	1.71	107	6.29	0.62	40	3.15	0.41	< 0.001

Abbreviations: AuDHD, cooccurring autism and ADHD; EL, elevated likelihood; LL, low likelihood.

^a^ *p*‐value from Kruskal‐Wallis test.

^b^ Monthly income household; range 1–7 (1 = < 20 K, 2 = 20–30 K, 3 = 30–40 K, 4 = 40–50 K, 5 = 50–60 K, 6 = 60–70 K, 7 = > 70 K [SEK]).

^c^ Education level; range: 1–4 (1 = primary, 2 = secondary, 3 = tertiary—Undergraduate, 4 = tertiary—Postgraduate).

^d^ Mullen scales of early learning (MSEL)—Composite score.

^e^ Autism diagnostic observation schedule‐2 (ADOS‐2)—Sum comparison score.

### 2.2 Sleep Measures

Caregiver‐rated infant sleep behaviors were assessed using the 34‐item Sleep and Settle Questionnaire (SSQ) (Matthey 2001) when infants were aged 10 and 14 months. Outcome measures were the number of awakenings during the night and the duration until settled (in minutes) during the night (average across evening and sleep). Evening is defined as the time between 6 pm and 10 pm and night is defined as the time between 10 pm and 5 am. Parent ratings were based on an average rating for the last week. Answers to the questions of interest were open‐ended. Reports of ranges were transformed to the average; settle durations of “a few minutes” were transformed to 2 min, and any ambiguous answers were excluded from further analysis.

Caregiver‐reported sleep behaviors at 24 and 36 months were assessed using the 33‐item Children's Sleep Habits Questionnaire (CSHQ) (Owens et al. 2000). Although conceptualized for an age range between four and 10 years, previous studies have shown its usefulness in younger ages as well as neurodivergent populations (Goodlin‐Jones et al. 2008), but see (Steur et al. 2017). Sleep behaviors are assessed for a typical week and their occurrence is rated on a 3‐point scale: usually (5–7 times/week), sometimes (2–4 times/week), and rarely (0–1 time/week). Items are grouped into eight subscales by building a sum score (Bedtime Resistance, Sleep Onset Delay, Sleep Duration, Sleep Anxiety, Night Wakings, Parasomnias, Sleep Disordered Breathing, Daytime Sleepiness). A “Total Sleep Disturbance” score is calculated by summing all 33 items. Parents further reported their child's total sleep duration during a 24‐h period in hours and minutes. Only participants with less than 20% missing data were included in the analysis. For these, missing values were imputed calculating the average of the respective subscale (Larsson et al. 2024; Waumans et al. 2010).

### 2.3 Diagnostic Assessment

At the 36‐month visit, which included several in‐person assessments, a clinical best estimate diagnosis according to DSM‐5 criteria (American Psychiatric Association 2013) was established by two experienced clinicians of whom one has met the child. One was a clinical licensed psychologist with several years of experience in the evaluation of neurodevelopmental conditions in different ages. The second (SB) was a clinical neuropsychologist, professor of child and adolescent psychiatry and expert regarding the assessment and intervention in the field of autism, ADHD and other neurodevelopmental conditions. Diagnostic decisions were supported by observation of symptoms during the visit including both unstructured and structured situations. Standardized diagnostic instruments included the Autism Diagnostic Observation Schedule 2nd edition (ADOS‐2) (Lord et al. 2012) and the Autism Diagnostic Interview‐Revised (ADI‐R) (Rutter et al. 2003) for autism, the ADHD rating scale (DuPaul et al. 2016) and the ADHD section of a structured diagnostic interview (The Preschool Age Psychiatric Assessment, Egger and Angold 2004) for ADHD, and the Child Behavior Checklist and the Caregiver‐Teacher Report Form for Ages 1.5–5 (CBCL 1.5‐5 and C‐TRF 1.5‐5) (Achenbach and Rescorla 2001) for externalizing (covering also attention problems), internalizing and overall problem behaviors. Participants did not need to meet a specific threshold in any available instrument for a diagnosis of ADHD; rather all available information was integrated qualitatively by the clinical team. The diagnostic decision required evidence from at least two sources (e.g., direct observation, information from parents) and two contexts (e.g., at the lab, at home). A diagnosis of ADHD was always given with the specifier “provisional” indicating that it was not required that all ADHD criteria were met but entailed that, given the available information and considering the child's age, the clinical team agreed that this classification was reasonable. Furthermore, this decision entailed that the diagnostic team expected that the child would fulfill diagnostic criteria for ADHD according to DSM‐5 criteria at a later timepoint. Certainty of the diagnostic judgment was rated on a 3‐point scale (low—medium—high). While for participants entering the study at an early stage, only an overall certainty rating (not separately for autism and ADHD) is available, certainty ratings regarding the diagnostic decision were provided separately for autism and ADHD later on (see Table S1 for certainty ratings within clinical groups). Furthermore, the degree of agreement regarding the diagnostic judgment was documented (low—medium—high) and showed overall very high consensus among the two clinicians. In 96% of the cases the agreement was high, in 4% it was medium and there were no cases with low agreement.

The Mullen Scales of Early Learning (MSEL) (Mullen 1995) were used to assess the children's developmental level. Further available information from the 36‐month visit included the SWAN rating scale (Swanson et al. 2012), the Early Childhood Behavior Questionnaire (ECBQ) (Putnam et al. 2006), the Vineland Adaptive Behavior Scale, Second Edition (Vineland‐II) (Sparrow et al. 2012), and video recorded parent–child interactions.

For four participants, ADOS‐2 scores were not available for the 36‐month visit. For these participants, available ADOS‐2 scores from the 24‐month visit were used for the analysis.

### 2.4 Statistical Analysis

All statistical analyses were done in R version 4.5.2 (R Core Team 2024) using RStudio 2025.09.2. For each sleep measure separate linear mixed‐effects models including the fixed effects group and time as well as their interaction and random intercepts for participants were used. Group contrasts comprised the comparisons of diagnostic with non‐diagnostic groups (−1/3 EL‐autism, −1/3 EL‐ADHD, −1/3 EL‐AuDHD, ½ EL‐none, ½ LL), EL diagnostic with EL non‐diagnostic groups (−1/3 EL‐autism, −1/3 EL‐ADHD, −1/3 EL‐AuDHD, 1 EL‐none, 0 LL), ADHD and autism with AuDHD (−1/2 EL‐autism, −1/2 EL‐ADHD, 1 EL‐AuDHD, 0 EL‐none, 0 LL), and autism with ADHD (−1 EL‐autism, 1 EL‐ADHD, 0 EL‐AuDHD, 0 EL‐none, 0 LL). Time was coded as 0 and 1 for both, comparisons of 10 and 14 months for the SSQ and 24 and 36 months for the CSHQ. Please note, for consistency the weights of the contrasts differ from the pre‐registration, which does not affect *t*‐ and *p‐*values, just estimates and standard errors. All models included the participants' sex as covariate. Although we originally planned to control for socioeconomic status as well, this was not done within the main models because data was missing (caregivers indicating they “don't know” or leaving the question empty) from 15 participants on monthly household income, eight participants on the mother's highest education level and 29 participants on the father's highest education level. Importantly, groups did not differ in monthly household income (*p* = 0.59) or paternal education level (*p* = 0.27). However, mothers of participants with ADHD had a lower education level than those from the groups without a diagnosis (all *p* < 0.04) and mothers from autistic participants had a lower educational level than mothers from participants of the low‐likelihood group (*p* = 0.03, see also Table 1). Thus, each model was re‐run controlling for maternal education level. Results of this sensitivity analysis are only reported in case it altered the result of the main model.

There were four participants with an uncertain diagnosis of autism due to difficulties in determining whether DSM‐5 criterion D (i.e., symptoms cause clinical impairment) was met at a young age (Zander and Bölte 2015). Thus, each model was additionally re‐run, excluding those four participants. In case exclusion modified results, this is mentioned correspondingly. Similarly, each model was re‐run, including the composite score of the MSEL. Changes in model output are reported accordingly.

The *lme4* and *lmerTest* libraries (Bates et al. 2015; Kuznetsova et al. 2017) were used for all models except sleep onset delay, sleep duration score and sleep disordered breathing, where cumulative link mixed models for ordinal data using the *ordinal* library (Christensen 2015) were calculated due to the distribution of the data. Group contrasts and interactions between group and time were inspected using the *emmeans* package (Lenth 2023). The following transformations of the data were carried out to improve model fit and ensure model assumptions are met: Duration until settled was square root transformed. For the analysis of the sleep disordered breathing score, the AuDHD group was removed due to almost no variation in responses (lowest possible score for all participants at 36 months and a change from 24 to 36 months in only two participants). Furthermore, scores > 5 (*n* = 4) were transformed to 5 leaving three score types (3 = no problems, 4 = little problems, 5 = some problems). Similarly, sleep duration scores were transformed to three ordered categories to improve model fit.

For an explorative analysis of associations between changes in sleep behaviors and dimensional measures of autistic and ADHD‐like traits, as well as externalizing and internalizing problems at 36 months, linear regressions were used. The difference between the two assessment time points was calculated for each sleep measure. Each model included the following predictors: ADOS‐2 comparative sum score, ADHD rating scale sum score, externalizing and internalizing behaviors from the CBCL, and the participant's sex. Due to assumption violation, models on sleep onset delay and sleep disordered breathing were calculated using ordinal regressions with the *ordinal* library (Christensen 2015). Additionally, change scores for sleep disordered breathing were transformed to three ordered categories (−1, 0, 1).

All plots were produced using the R library *ggplot2* (Hadley 2016). Plots depict the estimated marginal means and standard errors obtained from the *emmeans* package. Only for visualization purposes, estimates from models with ordered categorical outcomes were displayed using *mode = “mean.class*,*”* which expresses model predictions as the expected ordinal response category and not the original measurement unit.

## 3 Results

### 3.1 Infancy (10–14 Months)

The number of participants that went into each analysis and means and standard errors for all sleep behaviors assessed at 10 and 14 months are reported in Table 2. Number of nighttime awakenings and settle duration which were assessed at age 10 and 14 months did not differ between groups and assessment time points, nor was there an interaction between group and time of assessment (see also Table S2).

**TABLE 2:** Means and standard errors of sleep behaviors assessed at 10 and 14 months.

	EL‐ADHD			EL‐Autism			EL‐AuDHD			EL‐none			LL‐none		
	*n*	*M*	SEM	*n*	*M*	SEM	*n*	*M*	SEM	*n*	*M*	SEM	*n*	*M*	SEM
Number of night awakenings															
10 months	14	2.50	0.51	15	2.93	0.46	7	3.00	0.87	68	2.49	0.19	24	2.54	0.43
14 months	13	2.31	0.40	16	3.13	0.65	8	2.06	0.56	78	2.69	0.24	24	2.13	0.27
Settle duration (min)															
10 months	14	13.43	2.68	15	13.13	2.36	7	14.93	6.23	72	14.45	1.28	24	10.88	1.51
14 months	14	22.66	5.76	16	20.06	3.65	8	11.63	3.49	80	16.06	1.49	24	11.58	1.14

Abbreviations: AuDHD, cooccurring autism and ADHD; EL, elevated likelihood; LL, low likelihood.

### 3.2 Toddlerhood (24–36 Months)

The number of participants that went into each analysis and means and standard errors for sleep behaviors assessed at 24 and 36 months are reported in Table 3. Model outputs for sleep behaviors assessed at 24 and 36 months are reported in Tables S3 and S4. As mentioned in the introduction, we hypothesized that participants with a diagnosis (autism, ADHD, AuDHD) would differ from participants without a diagnosis (EL‐none, LL‐none) regarding the change in sleep behaviors from 24 to 36 months. This was not the case for any of the 10 investigated sleep behaviors using the CSHQ. Instead, participants with autism, ADHD or AuDHD differed from participants without a diagnosis regarding several sleep behaviors independent of age (see below).

**TABLE 3:** Means and standard errors of sleep behaviors assessed at 24 and 36 months.

	EL‐ADHD			EL‐Autism			EL‐AuDHD			EL‐none			LL‐none		
	*n*	*M*	SEM	*n*	*M*	SEM	*n*	*M*	SEM	*n*	*M*	SEM	*n*	*M*	SEM
Sleep duration (hrs)															
24 months	18	12.14	0.36	19	11.55	0.23	8	11.69	0.66	74	11.76	0.14	28	11.83	0.19
36 months	17	10.85	0.25	27	10.61	0.27	8	10.75	0.33	98	10.87	0.12	37	10.95	0.14
Sleep duration score															
24 months	18	4.42	0.38	19	3.61	0.20	8	4.00	0.42	75	3.45	0.11	28	3.68	0.19
36 months	19	4.44	0.34	27	4.05	0.36	8	4.13	0.67	99	3.88	0.13	35	3.51	0.15
Bedtime resistance															
24 months	18	11.44	0.70	19	12.83	0.66	8	10.38	1.13	75	10.94	0.38	28	10.00	0.57
36 months	19	11.68	0.74	27	10.99	0.55	8	10.63	1.13	99	11.39	0.28	35	9.93	0.42
Daytime sleepiness															
24 months	18	10.93	0.47	19	12.30	0.72	8	11.77	1.06	75	11.69	0.27	28	11.75	0.32
36 months	19	13.21	1.02	27	13.88	0.67	8	13.50	1.35	99	13.01	0.35	35	12.66	0.45
Sleep anxiety															
24 months	18	6.94	0.48	19	7.14	0.36	8	6.50	0.54	75	6.36	0.18	28	5.96	0.28
36 months	19	6.95	0.47	27	6.54	0.30	8	7.38	0.71	99	6.87	0.17	35	6.37	0.26
Sleep onset delay															
24 months	18	1.67	0.18	19	2.05	0.20	8	1.88	0.30	75	1.57	0.09	28	1.57	0.13
36 months	19	1.79	0.16	27	2.00	0.17	8	1.75	0.31	99	1.71	0.08	35	1.49	0.12
Parasomnias															
24 months	18	9.90	0.48	19	9.31	0.47	8	10.63	0.80	75	8.89	0.19	28	8.62	0.33
36 months	19	9.58	0.34	27	8.85	0.36	8	9.75	0.62	99	9.02	0.18	35	8.49	0.27
Night wakings															
24 months	18	5.89	0.39	19	5.95	0.49	8	5.25	0.59	75	5.53	0.22	28	5.57	0.37
36 months	19	5.79	0.46	27	4.93	0.34	8	5.88	0.55	99	5.41	0.19	35	5.77	0.30
Sleep disordered breathing															
24 months	18	3.61	0.18	19	3.37	0.27	8	3.25	0.16	75	3.23	0.07	28	3.18	0.09
36 months	19	3.32	0.22	27	3.41	0.14	8	3.00	0.00	99	3.20	0.05	34	3.18	0.08
Total sleep disturbance score															
24 months	18	49.78	1.52	19	52.70	1.93	8	49.77	2.39	75	47.58	0.88	28	46.51	1.21
36 months	19	52.05	1.95	27	51.20	1.47	8	51.75	2.74	99	50.23	0.80	34	47.14	1.11

Abbreviations: AuDHD, cooccurring autism and ADHD; EL, elevated likelihood; LL, low likelihood.

#### 3.2.1 Subtle Indicators of More Sleep Problems in Individuals With a Diagnosis, Independent of Age

**FIGURE 1:**
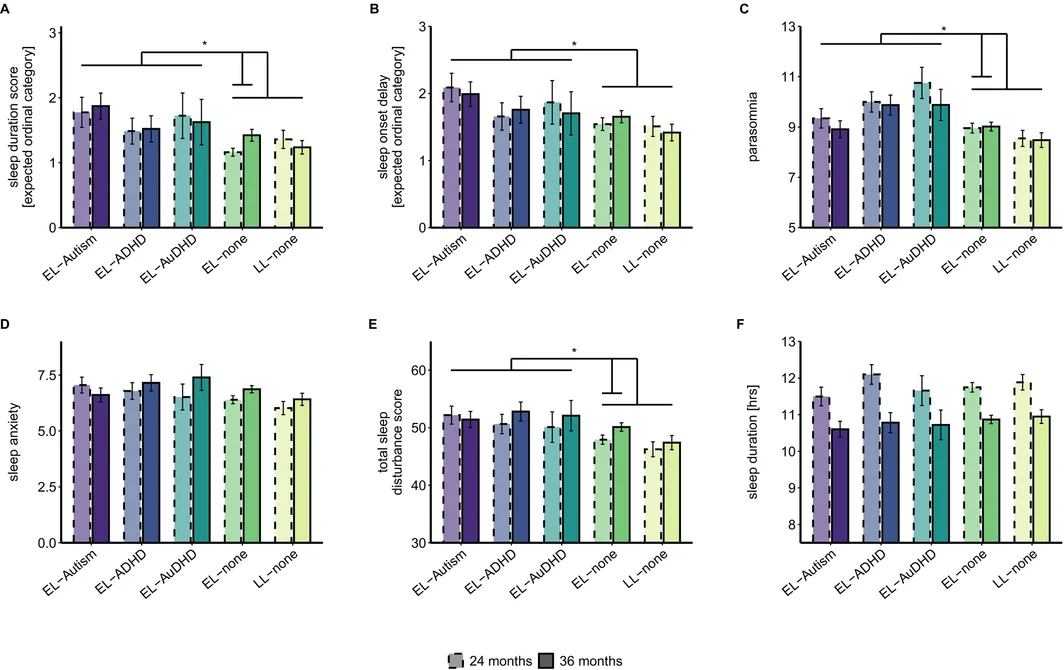
Sleep duration score (A), sleep onset delay (B), parasomnia score (C), sleep anxiety (D), total sleep disturbance score (E) and sleep duration in hours (F) separately for participants with autism, ADHD, cooccurring autism and ADHD (AuDHD), elevated likelihood of autism and/or ADHD and no diagnosis, low likelihood and no diagnosis at 24 (transparent, dashed line) and 36 months (filled, solid line). Plots depict the model‐estimated marginal means and standard errors. For ordered categorical outcomes, estimates are displayed as expected ordinal response category and not values in the original measurement unit. Annotations above the plots indicate significant planned group contrasts comparing the average of diagnostic groups (EL‐autism, EL‐ADHD, EL‐AuDHD) with the average of non‐diagnostic groups (EL‐none, LL‐none), and diagnostic groups with EL‐none. *p < 0.05.

Participants with a diagnosis had a higher sleep duration score (reflecting whether caregivers perceive their child's sleep duration as sufficient and consistent) than both participants at elevated likelihood of autism and/or ADHD without a diagnosis (OR = 0.23, log‐odds = −1.40, SE = 0.52, 95% CI [−2.42, −0.39], *z* = −2.71, *p* = 0.007) and all participants without a diagnosis (OR = 0.25, log‐odds = −1.46, SE = 0.53, 95% CI [−2.50, −0.42], *z* = −2.75, *p* = 0.006; Figure 1A). Adding the composite score of the MSEL to the model reduced the difference between diagnostic and non‐diagnostic groups (OR = 0.33, *p* = 0.126 and OR = 0.40, *p* = 0.051, respectively).

Further differences were found for sleep onset delay where scores were higher in participants with autism, ADHD or AuDHD compared to those without a diagnosis (OR = 0.44, log‐odds = −0.82, SE = 0.34, 95% CI [−1.50, −0.15], *z* = −2.40, *p* = 0.016, Figure 1B). Furthermore, participants with a diagnosis had a higher parasomnia score than participants without a diagnosis (std. *β* = −0.60, *b* = −1.04, SE = 0.28, 95% CI [−1.59, −0.49], *t*(182) = −3.73, *p* < 0.001) and participants at elevated likelihood without a diagnosis (std. *β* = −0.46, *b* = −0.81, SE = 0.28, 95% CI [−1.36, −0.25], *t*(180) = −2.85, *p* = 0.005, Figure 1C). When controlling for the cognitive level, this pattern of results remained the same but additionally, autistic toddlers showed lower parasomnia scores than those with ADHD (std. *β* = 0.55, *b* = 0.96, SE = 0.46, 95% CI [0.06, 1.87], *t*(192) = 2.09, *p* = 0.038).

There was a trend for a higher sleep anxiety score in participants with autism and/or ADHD compared to participants without a diagnosis (std. *β* = 0.30, *p* = 0.067, Figure 1D). This was slightly more pronounced when excluding the four participants with autism with an uncertain diagnosis (std. *β* = −0.33, *b* = −0.54, SE = 0.27, 95% CI [−1.06, −0.01], *t*(188) = −2.01, *p* = 0.046) as well as when controlling for maternal educational level (std. *β* = −0.36, *b* = −0.59, SE = 0.27, 95% CI [−1.13, −0.05], *t*(185) = −2.17, *p* = 0.031).

Lastly, individuals with a diagnosis showed a higher total sleep disturbance score than both individuals at elevated likelihood of autism and/or ADHD without a diagnosis (std. *β* = −0.34, *b* = −2.52, SE = 1.26, 95% CI [−5.02, −0.03], *t*(194) = −1.995, *p* = 0.047) and all participants without a diagnosis (std. *β* = −0.49, *b* = −3.62, SE = 1.25, 95% CI [−6.09, −1.15], *t*(195) = −2.89, *p* = 0.004, Figure 1E). This difference was less pronounced when controlling for the cognitive level (std. *β* = −0.35, *p* = 0.015 and std. *β* = −0.49, *p* = 0.062 respectively).

#### 3.2.2 Age Effects Independent of Diagnostic Status

In addition to the reported higher total sleep disturbance score in individuals with a diagnosis compared to those without a diagnosis, there was an increase in the total score from 24 to 36 months across groups (std. *β* = −0.18, *b* = −1.34, SE = 0.64, 95% CI [−2.61, −0.08], *t*(142) = −2.11, *p* = 0.037).

**FIGURE 2:**
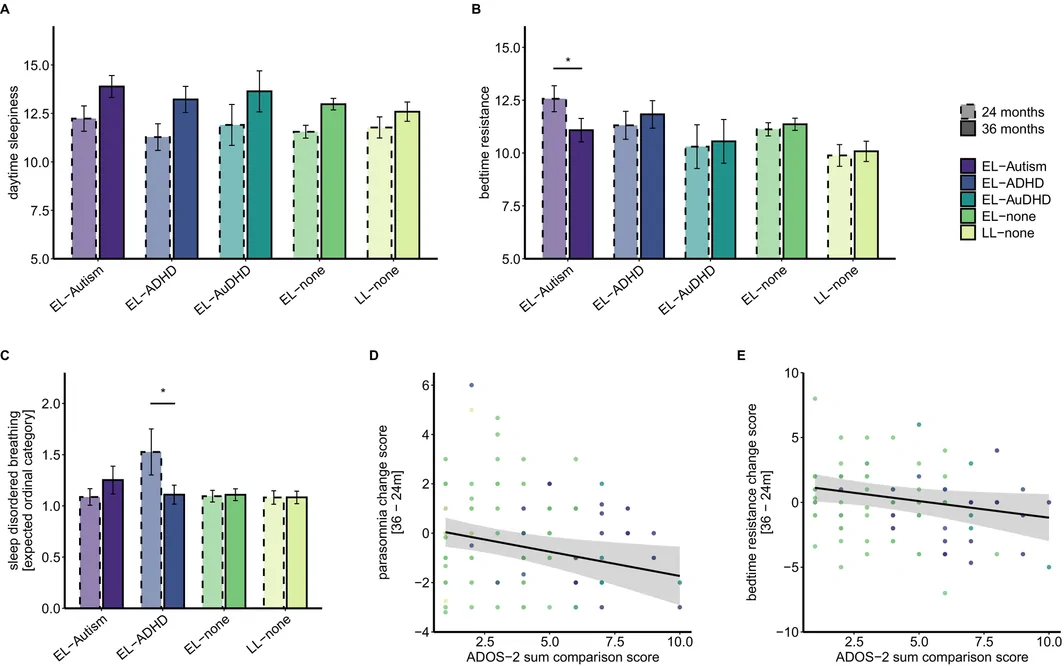
Daytime sleepiness (A), bedtime resistance (B) and sleep disordered breathing (C) separately for participants with autism, ADHD, cooccurring autism and ADHD (AuDHD), elevated likelihood of autism and/or ADHD and no diagnosis, low likelihood and no diagnosis, at 24 (transparent, dashed line) and 36 months (filled, solid line). Association between change in parasomnia score from 24 to 36 months (D) and ADOS‐2 sum comparison score. Association between change in bedtime resistance from 24 to 36 months (E) and ADOS‐2 sum comparison score. A–C: Plots depict the model‐estimated marginal means and standard errors. For ordered categorical outcomes, estimates are displayed as expected ordinal response categories and not values in the original measurement unit. D and E: Regression lines depict model‐based predicted values and the shaded area the 95% confidence interval. Points represent the raw observed values.

Furthermore, sleep duration reported in hours decreased from 24 to 36 months (std. *β* = 0.87, *b* = 0.99, SE = 0.13, 95% CI [0.73, 1.26], *t*(148) = 7.39, *p* < 0.001, Figure 1F), while daytime sleepiness scores increased from 24 to 36 months (std. *β* = −0.51, *b* = −1.51, SE = 0.32, 95% CI [−2.14, −0.88], *t*(146) = −4.77, *p* < 0.001, Figure 2A). Sleep anxiety increased from 24 to 36 months (std. *β* = −0.20, *b* = −0.33, SE = 0.17, 95% CI [−0.66, −0.005], *t*(148) = −2.01, *p* = 0.047).

#### 3.2.3 Differential Trajectories of Bedtime Resistance and Sleep Disordered Breathing Among Diagnostic Groups

Changes in bedtime resistance and sleep disordered breathing scores from 24 to 36 months differed between participants with autism and ADHD. While autistic participants had a greater decrease in the bedtime resistance score from 24 to 36 months than children with ADHD (autism vs. ADHD × time interaction: std. *β* = 0.69, *b* = 2.01, SE = 0.79, 95% CI [0.44, 3.57], *t*(153) = 2.53, *p* = 0.012, Figure 2B), sleep disordered breathing scores decreased more in the ADHD than the autism group from 24 to 36 months (autism vs. ADHD × time interaction: OR = 0.04, log‐odds = 2.24, SE = 1.14, 95% CI [−5.93, −0.47], *z* = −2.30, *p* = 0.022, Figure 2C). To get a more comprehensive picture of the two interactions, simple slopes and pairwise comparisons were calculated.

This confirmed a significant decrease in bedtime resistance scores from 24 to 36 months in the autism group (*t*(156) = −2.55, *p* = 0.007), with no significant changes across time in the other groups (all *p* > 0.37). Bedtime resistance scores were greater in the autism than the two groups without a diagnosis at 24 months (all *p* < 0.04) but not at 36 months (all *p* > 0.17). Participants with ADHD had slightly higher bedtime resistance scores than the low likelihood group without a diagnosis at 36 months (*t*(275) = 2.16, *p* = 0.032) but not at 24 months (*p* = 0.091) and they further did not differ from any other group at any time point (all *p* > 0.29).

Sleep disordered breathing scores significantly decreased from 24 to 36 in participants with ADHD (*z* = −2.07, *p* = 0.04), with no further significant changes across the one‐year interval within any other group (all *p* > 0.19). Individuals with ADHD had a higher sleep disordered breathing score than the two groups without a diagnosis (all *p* < 0.026) and the autism group (*p* = 0.049) at 24 months but not at 36 months (all *p* > 0.34).

### 3.3 Associations Between Changes in Sleep Behaviors and Dimensional Clinical Measures

The explorative analyses of associations between changes in sleep behaviors and dimensional measures of autism and ADHD as well as internalizing and externalizing problem behaviors, revealed statistically significant associations with only two sleep measures. Output from all models is reported in Tables S5–S7. A greater ADOS‐2 sum comparison score at 36‐months was associated with a greater decrease in the parasomnia score from 24 to 36 months in the whole sample (std. *β* = −0.29, *b* = −0.20, SE = 0.08, 95% CI [−0.35, −0.05], *t*(103) = −2.61, *p* = 0.011) and when only considering participants at elevated likelihood of autism and/or ADHD (std. *β* = −0.26, *b* = −0.17, SE = 0.08, 95% CI [−0.33, −0.01], *t*(84) = −2.16, *p* = 0.034, Figure 2D). A similar pattern was found for changes in bedtime resistance (Figure 2E), where greater ADOS‐2 sum comparison scores at 36‐months were associated with a greater decrease in the bedtime resistance score from 24 to 36 months, which was only significant in participants at elevated likelihood of autism and/or ADHD (std. *β* = −0.25, *b* = −0.26, SE = 0.12, 95% CI [−0.49, −0.02], *t*(84) = −2.14, *p* = 0.035) but not across the whole sample (std. *β* = −0.20, *b* = −0.20, SE = 0.11, 95% CI [−0.42, 0.01], *t*(103) = −1.85, *p* = 0.067). Please note, however, these associations are based on a smaller sample due to the assessment of the ADHD rating scale starting at a later time point during the study (see also Table 1).

To better understand these associations, we calculated zero‐order correlations between the dimensional measures (assessed at 36 months) and the sleep behaviors separately at 24 and 36 months (analyses not pre‐registered). These revealed a significant but weak positive association between the parasomnia score at 24 months and the ADOS‐2 sum comparison score but no association with the 36‐month parasomnia score or the bedtime resistance scores with the ADOS‐2 sum comparison score (see Table S8). Externalizing and internalizing symptoms assessed at 36 months were most consistently associated with the total sleep disturbance score and the sleep duration score at both 24 and 36 months.

## 4 Discussion

This study aimed to investigate whether individuals who were diagnosed with either autism, ADHD, or cooccurring autism and ADHD (AuDHD) at 3 years differ in their early sleep trajectories from individuals with or without an elevated familial likelihood of autism and/or ADHD who did not meet diagnostic criteria. While we could not confirm differential sleep *trajectories* in infancy and toddlerhood in individuals with autism and/or ADHD compared to those without a diagnosis, five of the twelve investigated sleep behaviors indicated more sleep problems in diagnosed individuals. This difference was subtle and concerned the sleep onset delay, sleep anxiety, parasomnias, how sufficient and consistent sleep duration was perceived, and overall total sleep disturbance. Besides an improvement in bedtime resistance from 24 to 36 months unique to autistic children, there was little indication that a specific sleep behavior is linked to only one diagnostic group. Together, our results suggest sleep associations with neurodevelopmental conditions to reflect overall more transdiagnostic than diagnosis‐specific processes during toddlerhood, although differences between individuals with and without a diagnosis are small at this age.

At age 10 and 14 months we used the SSQ (Matthey 2001) to assess the number of nighttime awakenings and the duration until the infant is settled at night. Both measures did not indicate any group‐specific changes from 10 to 14 months or a general difference between participants that were diagnosed with autism and/or ADHD at 3 years compared to those who did not meet diagnostic criteria. Previous longitudinal studies on infant sleep in individuals later diagnosed with autism or ADHD present a mixed picture showing indicators of more sleep problems in individuals with a diagnosis at specific time points (Begum‐Ali et al. 2023; MacDuffie et al. 2020; Scott et al. 2013). Discrepancies could partly be explained by the use of different sleep parameters in these studies. Additionally, the inter‐ and intraindividual variability of infant sleep is generally very high (Iglowstein et al. 2003; Schoch et al. 2020), suggesting that infant sleep (problems) will unlikely function as an early marker of neurodevelopmental conditions. Nonetheless, further studies using comparable measures of sleep are needed for a more comprehensive evaluation of infant sleep in individuals later diagnosed with autism and/or ADHD.

### 4.1 Transdiagnostic Effects

Sleep behaviors at 24 and 36 months were assessed using the Children's Sleep Habits Questionnaire (Owens et al. 2000). Across the 10 sleep behaviors examined, caregivers reported more sleep problems in five domains among children who later received a diagnosis of autism and/or ADHD compared to children without a diagnosis. These findings suggest that sleep difficulties are already detectable in toddlerhood in children with neurodevelopmental conditions, but that they are not expressed as a uniform elevation across all aspects of sleep.

Some sleep‐related differences were less robust after accounting for cognitive ability. Differences regarding the total sleep disturbance score, sleep onset delay and sleep duration score either decreased when controlling for cognitive ability or only differentiated individuals with and without a diagnosis without clearly separating elevated likelihood children who did versus did not later receive a diagnosis of autism and/or ADHD. Furthermore, differences regarding sleep anxiety were a trend. This pattern suggests that sleep difficulties associated with initiating sleep (i.e., increased sleep onset delay and sleep anxiety) may reflect broader developmental factors, including cognitive and regulatory capacities, rather than diagnostic status alone.

In contrast, parasomnia‐related behaviors showed the most consistent association with later autism and/or ADHD diagnosis. This is in line with previous questionnaire‐based as well as polysomnographic studies showing a higher prevalence of parasomnias in neurodevelopmental conditions (see (Bruni et al. 2025) for a review). While parasomnias can include a wide range of different sleep‐associated behaviors like sleep talking and walking, sleep terrors, confusional arousals, and enuresis, caregivers of children with autism and/or ADHD largely reported more frequent awakenings, screaming, sweaty, and inconsolable as well as more restlessness during sleep. Parasomnia‐related behaviors may therefore represent an aspect of sleep in which atypical arousal regulation, sleep‐stage transitions, or broader neurodevelopmental vulnerability are already apparent early in life.

Although total daily sleep duration was comparable among participants, caregivers perceived the duration and consistency of their child's sleep duration as more problematic in children with autism and/or ADHD. This distinction is important because it suggests that sleep health in neurodevelopmental conditions may not be captured fully by average sleep duration alone. While most research on associations between sleep and developmental and health outcomes was done with regards to sleep duration (Chaput et al. 2017), the variability of sleep wake patterns might be even more important for health and development (Becker et al. 2017; Sletten et al. 2023). In line with our findings, actigraphy‐based studies have shown higher night‐to‐night variability in sleep patterns in neurodivergent children and adolescents (Anders et al. 2011; Gruber et al. 2000; Langberg et al. 2019), highlighting that it is insufficient to merely assess average sleep duration and ask about the “typical” week (Langberg et al. 2019).

It might also be the case that the caregiver‐based reports of sleep are biased, as the broader pediatric sleep literature has repeatedly shown incomplete convergence between caregiver‐report and objective measures of sleep (e.g., Adams et al. 2019; Perpétuo et al. 2020; Werner et al. 2008). The concordance of subjective and objective measures might be dependent on context. Overestimation of sleep duration and underestimation of nighttime awakenings are common and might be more pronounced the older the child and the smaller the involvement of the caregiver in the sleep routine which might depend on the self‐soothing abilities of the child (Rudzik et al. 2018; Tikotzky and Volkovich 2019). Accordingly, concordance of objective and caregiver‐rated subjective measures of sleep has been shown to be higher in neurodivergent children compared to their neurotypical peers—a finding which might be explained by increased co‐sleeping or higher demands due to sleep problems in neurodivergent children (Pinghini et al. 2025). Overall, discordance between objective and subjective measures of sleep in neurodevelopmental conditions mirrors those seen in neurotypicals, although different contexts influencing this pattern need to be investigated (O'Sullivan et al. 2023).

Potential discrepancies between caregiver‐report and objective measures of sleep do not necessarily undermine the relevance of caregiver reports. Our findings suggest that what is perceived as the optimal amount of sleep might differ between neurodivergent and neurotypical populations. This may indicate that caregiver‐report measures capture aspects of sleep that are clinically meaningful to families, such as bedtime stress, night waking, irregularity, or daytime consequences. In autistic toddlers, sleep problems were associated with maternal stress independently of autism symptom severity and more problematic sleep‐related cognitions were associated with more disrupted sleep across the whole sample of neurotypical and autistic toddlers (Levin and Scher 2016). This highlights the need to educate parents about sleep and investigate sleep in the context of the whole family. Finally, comparing subjective and objective sleep assessments is essential for distinguishing between differences in sleep physiology, differences in sleep‐related family burden, and differences in how sleep problems are perceived and reported.

Overall, sleep differences during toddlerhood were less prominent and pertained to specific sleep behaviors compared to cross‐sectional studies in children where more sleep problems have been observed in a wide range of sleep behaviors in children with autism or ADHD (Cortese et al. 2009; Díaz‐Román et al. 2018). This developmental pattern might suggest that sleep difficulties become more differentiated, more impairing, or more detectable with age as environmental demands increase and behavioral regulation becomes more central to daily functioning. The general high level of sleep problems across the whole sample in the current study is in line with previous studies in pre‐school children using the same sleep questionnaire (Goodlin‐Jones et al. 2008), suggesting a generally higher level of sleep problems in younger children (Williamson et al. 2019). Thus, a time span longer than 1 year might be necessary to unveil differential trajectories of sleep behaviors in individuals with and without a diagnosis of autism and/or ADHD.

### 4.2 Diagnosis Specific Effects

We found that individuals on the autism spectrum and with ADHD significantly differed in their changes of sleep disordered breathing and bedtime resistance from 24 to 36 months. This is in contrast to the transdiagnostic effects reported above where subtle differences across toddlerhood were observed between individuals with and without a diagnosis.

Results indicated a decrease in symptoms related to sleep disordered breathing in individuals with ADHD. This result should be interpreted with considerable caution because the scores in this subscale showed limited variability and were generally low across the sample, suggesting that the observed differences are unlikely to reflect clinically significant sleep disordered breathing. Future longitudinal studies incorporating objective measures of respiratory functioning during sleep are necessary to evaluate whether children with ADHD indeed show different trajectories of symptoms related to sleep disordered breathing compared to other neurodivergent as well as neurotypical individuals.

None of our results indicated a significant difference between individuals with AuDHD compared to the combined group of individuals with either autism or ADHD. Literature on sleep in AuDHD is scarce and limited to older ages than in the current study (e.g., Martinez‐Cayuelas et al. 2024; Thomas et al. 2018). To our knowledge, this is the first study to investigate sleep behaviors in AuDHD in infants and toddlers. The absence of statistically significant differences should not be interpreted as evidence of equivalent sleep profiles, given the small AuDHD group and limited power to detect group‐by‐time interactions. For example, night awakenings assessed with the CSHQ tended to increase from 24 to 36 months in AuDHD but decreased in the combined group of individuals with either autism or ADHD (std. *β* = 0.65, *p* = 0.09). This increase in the AuDHD group may be consistent with prior reports that cooccurring ADHD in autism is associated with aspects of sleep maintenance (Martinez‐Cayuelas et al. 2024). However, the present estimate was imprecise and did not provide strong evidence for a group specific trajectory. Future, better sized studies are necessary to determine whether despite a potential increased likelihood for sleep problems, young children with AuDHD show comparable sleep behaviors compared to individuals with autism or ADHD.

Our better powered dimensional approach of investigating associations between changes in sleep behaviors with autistic and ADHD‐like traits as well as internalizing and externalizing behaviors revealed among children with an elevated likelihood of autism and/or ADHD that an improvement in bedtime resistance from 24 to 36 months was related to more autistic traits at 36 months. This is in line with our categorical approach, where bedtime resistance scores decreased from 24 to 36 months in the autism group with no changes in ADHD, AuDHD and toddlers without a diagnosis. Autistic individuals had a higher bedtime resistance score than those without a diagnosis at 24 months, which was not the case at 36 months. A similar pattern of results was seen in school‐aged children, where autistic children generally had more sleep problems than a comparison group and across a one‐year interval the autistic children but not the comparison group showed an improvement in bedtime resistance (Fletcher et al. 2017; May et al. 2015). One possible explanation is that families of children with more pronounced autistic traits increasingly implemented bedtime routines or environmental adaptations during the two assessment time points. Implementation of bedtime routines in pre‐school years has been found to be associated with less bedtime resistance, parental stress (Larsen et al. 2023) and overall better sleep (Mindell et al. 2015). Although this is highly speculative, increasing awareness of the child's needs might have led to the environmental adaptations.

A greater decrease in the parasomnia score from 24 to 36 months was also associated with more autistic traits at 3 years in the whole sample as well as in the sub‐sample of children with elevated familial likelihood of autism and/or ADHD. In line with this, May et al. (2015) found an improvement in the CSHQ parasomnia score in autistic school‐aged children. On an item level, across the whole sample, participants in our study showed greatest improvement across the one‐year interval regarding bed wetting, nightmares, and restlessness during sleep. This suggests that the CSHQ parasomnia score should not be interpreted as a uniform improvement across all parasomnia behaviors. Our findings could reflect maturational changes or changes in family routines, which might be specifically pronounced between 2 and 3 years in individuals with more autistic traits.

Finally, our additional correlational analyses suggest that, in this age range, parent‐reported sleep might be more closely related to internalizing and externalizing difficulties than to autistic and ADHD‐like traits per se. A growing body of research highlights the link between sleep problems and externalizing and internalizing problems in neurodivergent children (Reynolds et al. 2017; Thomas et al. 2018) as well as the general population (Quach et al. 2018; Williamson et al. 2021), with hints for a bi‐directional association between internalizing/depressive symptoms and sleep from toddlerhood to school‐age (Sivertsen et al. 2021).

The overall less diagnosis specific effects in our study could also reflect difficulties in the identification of early ADHD symptoms. Autism can be diagnosed with high reliability by 3 years of age (Ozonoff et al. 2015). In contrast, ADHD diagnoses are usually not given before school‐age, although ADHD‐related symptoms may emerge much earlier (Rocco et al. 2021) as seen in Miller et al. (2020) who identified an ADHD and autism class in 36‐month old toddlers with a family history of ADHD or autism. Consistent with this, Miller et al. (2025) found that ADHD symptoms assessed at 24 months showed moderate stability into the preschool period, and that children with later ADHD concerns already had elevated ADHD symptoms at age two. Work on early neurodevelopmental profiles in familial‐likelihood cohorts has shown only partial continuity from early childhood to later outcomes, indicating that early phenotypic presentations may change over development (Charman et al. 2026). In the current study, ADHD diagnoses were assigned with the specifier provisional. Both autism and ADHD classifications reflected the best‐estimate clinical judgment of the diagnostic team at 36‐month, and raters had a high degree of consensus. Nevertheless, the classifications assigned at that time point are not evidence of a definitive and stable diagnosis. Conversely, we cannot rule out that individuals without a diagnosis at 36 months might meet criteria for autism and/or ADHD at a later age, consistent with evidence that some children evaluated longitudinally from infancy received an autism diagnosis only after age five (Ozonoff et al. 2018). Despite this limitation, early best‐estimate classifications are valuable for characterizing emerging developmental pathways before diagnostic outcomes are fully consolidated.

## 5 Conclusion

To summarize, we found comparable sleep behaviors assessed at 10 and 14 months, while subtle differences were observed at 24 and 36 months with regard to a few specific sleep behaviors. These included a higher sleep disturbance, specifically longer sleep onset latencies, more sleep anxiety, more parasomnias, and caregivers perceiving their child's sleep duration as insufficient and inconsistent despite comparable sleep durations in individuals with autism and/or ADHD compared to those without a diagnosis of autism and/or ADHD. Specifically, in autistic individuals, bedtime resistance is high at 24 months but shows the greatest improvement across the one‐year interval. Overall, sleep behaviors appear more transdiagnostic than diagnosis‐specific during toddlerhood. Longitudinal studies assessing a broader range of comparable sleep behaviors including objective measures across infancy and early childhood are necessary to confirm and complement the picture portrayed here.

## Funding

This work was supported by Riksbankens Jubileumsfond, Stiftelsen Sunnerdahls Handikappfond, Knut och Alice Wallenbergs Stiftelse, Innovative Medicines Initiative 2 Joint Undertaking grant (777394), European Commission, H2020 project CANDY; grant (847818).

## Ethics Statement

Written informed consent was obtained from all caregivers. The Stockholm Regional Ethics Committee approved the study (2010/2085‐31/3).

## Conflicts of Interest

Sven Bölte declares no direct conflicts of interest related to this article. Bölte discloses that he has in the last 3 years acted as a consultant or lecturer for Medice, Takeda, Neuraxpharm, Merk Sharp & Dohme, and LinusBio. He receives royalties for textbooks and diagnostic tools from Hogrefe, UTB, Ernst Reinhardt, Kohlhammer, and Liber. Bölte is CEO of NeuroSupportSolutions International AB. The remaining authors declare no conflicts of interest.

## Supporting information


**Table S1:** Diagnostic certainty ratings.
**Table S2:** Linear mixed‐effects model predictors of sleep behaviors assessed with the Sleep and Settle Questionnaire.
**Table S3:** Linear mixed‐effects model predictors of sleep behaviors assessed with the Children's Sleep Habits Questionnaire.
**Table S4:** Cumulative link mixed model predictors of sleep behaviors assessed with the Children's Sleep Habits Questionnaire.
**Table S5:** Linear regression model predictors of changes in sleep behaviors from 10 to 14 months assessed with the Sleep and Settle Questionnaire.
**Table S6:** Linear regression model predictors of changes in sleep behaviors from 24 to 36 months assessed with the Children's Sleep Habits Questionnaire.
**Table S7:** Cumulative link model predictors of changes in sleep behaviors from 24 to 36 months assessed with the Children's Sleep Habits Questionnaire.
**Table S8:** Zero‐order correlations of dimensional measures of autism and ADHD and externalizing and internalizing problems with sleep behaviors at 24 and 36 months assessed with the Children's Sleep Habits Questionnaire.

## Data Availability

The analyses presented here were preregistered (https://osf.io/v8tdk/). The data and code necessary to reproduce the analyses presented here are not publicly accessible, but will be made available upon reasonable request to the corresponding author. Note that sharing of pseudonymized personal data will require a data sharing agreement according to Swedish and EU law.
