# Supplementary material for: Sleep in Young Children With Autism, ADHD and Combined Presentations
Source: Autism Res. 2026 Jul 25;19(9):e70326. doi: 10.1002/aur.70326 (PMC13599689; doi:10.1002/aur.70326)
Supplement: Supplementary file 1 [file AUR-19-0-s001.docx]

**Supporting Information**

**Sleep in young children with autism, ADHD and combined presentations**

Eva-Maria Kurz^1^, Sven Bölte^2,3,4^, Terje Falck-Ytter^1,2^

1 Development and Neurodiversity Lab, Department of Psychology, Uppsala University; Uppsala, Sweden

2 Center of Neurodevelopmental Disorders (KIND), Division of Neuropsychiatry, Department of Women’s and Children’s Health, Karolinska Institute, Stockholm, Sweden

3 Child and Adolescent Psychiatry, Stockholm Health Care Services, Region Stockholm, Stockholm, Sweden

4 Curtin Autism Research Group, Curtin School of Allied Health, Curtin University, Perth, WA Australia

**Table S1**

Diagnostic Certainty Ratings

|  |  | autism | |  | ADHD | |
| --- | --- | --- | --- | --- | --- | --- |
| group | certainty | n | proportion |  | n | proportion |
| EL-ADHD | low | 6 | 38 |  | 2 | 12 |
|  | medium | 7 | 44 |  | 12 | 71 |
|  | high | 3 | 19 |  | 3 | 18 |
| EL-AuDHD | low | 4 | 50 |  | 5 | 63 |
|  | medium | 2 | 25 |  | 3 | 38 |
|  | high | 2 | 25 |  | 0 | 0 |
| EL-autism | low | 5 | 17 |  | 9 | 43 |
|  | medium | 10 | 35 |  | 9 | 43 |
|  | high | 14 | 48 |  | 3 | 14 |

*Note.* Please note that these ratings reflect how certain the raters were regarding their diagnostic decision rather than the certainty that the respective diagnosis is present

**Table S2**

*Linear mixed-effects model predictors of sleep behaviors assessed with the Sleep and Settle Questionnaire*

|  |  | *Std coeff* | *coeff* | *SE* | *CI* | | *t* |
| --- | --- | --- | --- | --- | --- | --- | --- |
| DV | Predictor |  |  |  | *2.5%* | *97.5%* |  |
| # of night awakenings | Diagnosis vs. no diagnosis | -0.08 | -0.15 | 0.34 | 0.71 | 0.84 | -0.68 |
|  | EL diagnosis vs. EL no diagnosis | -0.02 | -0.04 | 0.34 | -0.71 | 0.62 | -0.12 |
|  | Autism and ADHD vs. AuDHD | -0.14 | -0.27 | 0.66 | -1.57 | 1.04 | -0.40 |
|  | Autism vs ADHD | -0.26 | -0.49 | 0.58 | -1.63 | 0.66 | -0.84 |
|  | Assessment time point | 0.10 | 0.19 | 0.21 | -0.23 | 0.60 | 0.37 |
|  | sex | 0.04 | 0.08 | 0.28 | -0.47 | 0.63 | 0.29 |
|  | Diagnosis vs. no diagnosis × time | 0.003 | 0.01 | 0.38 | -0.75 | 0.76 | 0.01 |
|  | EL diagnosis vs. EL no diagnosis × time | 0.13 | 0.26 | 0.38 | -0.50 | 1.02 | 0.68 |
|  | Autism and ADHD vs. AuDHD × time | -0.47 | -0.87 | 0.74 | -2.33 | 0.58 | -1.19 |
|  | Autism vs ADHD × time | -0.31 | -0.57 | 0.70 | -1.95 | 0.80 | -0.83 |
| Settle duration | Diagnosis vs. no diagnosis | -0.12 | -0.16 | 0.25 | -0.65 | 0.33 | -0.65 |
|  | EL diagnosis vs. EL no diagnosis | 0.04 | 0.05 | 0.24 | -0.43 | 0.53 | 0.22 |
|  | Autism and ADHD vs. AuDHD | -0.54 | -0.76 | 0.48 | -1.70 | 0.19 | -1.58 |
|  | Autism vs ADHD | 0.13 | 0.19 | 0.42 | -0.64 | 1.02 | 0.45 |
|  | Assessment time point | -0.20 | -0.28 | 0.17 | -0.62 | 0.06 | -1.62 |
|  | sex | 0.13 | 0.18 | 0.20 | -0.22 | 0.57 | 0.87 |
|  | Diagnosis vs. no diagnosis × time | -0.20 | -0.28 | 0.31 | -0.90 | 0.34 | -0.89 |
|  | EL diagnosis vs. EL no diagnosis × time | -0.20 | -0.28 | 0.31 | -0.90 | 0.34 | -0.89 |
|  | Autism and ADHD vs. AuDHD × time | -0.54 | -0.76 | 0.61 | -1.97 | 0.45 | -1.25 |
|  | Autism vs ADHD × time | 0.10 | 0.14 | 0.57 | -0.98 | 1.25 | 0.24 |

*Note.* EL = elevated likelihood; AuDHD = cooccurring autism and ADHD.

**Table S3**

*Linear mixed-effects model predictors of sleep behaviors assessed with the Children’s Sleep Habits Questionnaire*

|  |  | *Std coeff* | *coeff* | *SE* | *CI* | | *t* |
| --- | --- | --- | --- | --- | --- | --- | --- |
| DV | Predictor |  |  |  | *2.5%* | *97.5%* |  |
| Sleep duration (hrs) | Diagnosis vs. no diagnosis | 0.11 | 0.14 | 0.18 | -0.22 | 0.49 | 0.76 |
|  | EL diagnosis vs. EL no diagnosis | 0.07 | 0.08 | 0.28 | -0.27 | 0.44 | 0.45 |
|  | Autism and ADHD vs. AuDHD | -0.05 | -0.06 | 0.37 | -0.78 | 0.67 | -0.15 |
|  | Autism vs ADHD | 0.34 | 0.39 | 0.29 | -0.18 | 0.97 | 1.35 |
|  | **Assessment time point** | **0.87** | **0.99** | **0.13** | **0.73** | **1.26** | **7.39***** |
|  | sex | -0.11 | -0.12 | 0.14 | -0.40 | 1.61 | -0.85 |
|  | Diagnosis vs. no diagnosis × time | 0.12 | 0.14 | 0.25 | -0.34 | 0.63 | 0.58 |
|  | EL diagnosis vs. EL no diagnosis × time | 0.15 | 0.17 | 0.25 | -0.32 | 0.66 | 0.69 |
|  | Autism and ADHD vs. AuDHD × time | 0.15 | 0.17 | 0.49 | -0.81 | 1.14 | 0.34 |
|  | Autism vs ADHD × time | -0.37 | -0.42 | 0.42 | -1.25 | 0.41 | -1.00 |
| Total score | **Diagnosis vs. no diagnosis** | **-0.49** | **-3.62** | **1.25** | **-6.09** | **-1.15** | **-2.89**** |
|  | **EL diagnosis vs. EL no diagnosis** | **-0.34** | **-2.52** | **1.26** | **-5.02** | **-0.03** | **-2.00*** |
|  | Autism and ADHD vs. AuDHD | -0.09 | -0.67 | 2.62 | -5.83 | 4.51 | -0.25 |
|  | Autism vs ADHD | -0.01 | -0.08 | 2.00 | -4.02 | 3.87 | -0.04 |
|  | **Assessment time point** | **-0.18** | **-1.34** | **0.64** | **-2.61** | **-0.08** | **-2.11*** |
|  | sex | 0.19 | 1.39 | 1.00 | -0.58 | 3.36 | 1.39 |
|  | Diagnosis vs. no diagnosis × time | 0.07 | 0.55 | 1.18 | -1.78 | 2.88 | 0.47 |
|  | EL diagnosis vs. EL no diagnosis × time | 0.14 | 1.08 | 1.18 | -1.25 | 3.41 | 0.91 |
|  | Autism and ADHD vs. AuDHD × time | 0.17 | 1.29 | 2.30 | -3.27 | 5.84 | 0.56 |
|  | Autism vs ADHD × time | 0.39 | 2.93 | 2.00 | -1.02 | 6.88 | 1.46 |
| Bedtime resistance | Diagnosis vs. no diagnosis | -0.23 | -0.66 | 0.49 | -1.62 | 0.31 | -1.35 |
|  | EL diagnosis vs. EL no diagnosis | -0.01 | -0.03 | 0.50 | -1.01 | 0.94 | -0.07 |
|  | Autism and ADHD vs. AuDHD | -0.43 | -1.27 | 1.02 | -3.29 | 0.75 | -1.24 |
|  | Autism vs ADHD | -0.09 | -0.26 | 0.78 | -1.80 | 1.29 | -0.33 |
|  | Assessment time point | 0.02 | 0.06 | 0.25 | -0.44 | 0.55 | 0.23 |
|  | sex | -0.10 | -0.29 | 0.39 | -1.06 | 0.48 | -0.74 |
|  | Diagnosis vs. no diagnosis × time | 0.16 | 0.45 | 0.47 | -0.46 | 1.37 | 0.98 |
|  | EL diagnosis vs. EL no diagnosis × time | 0.16 | 0.48 | 0.47 | -0.45 | 1.40 | 1.02 |
|  | Autism and ADHD vs. AuDHD × time | 0.25 | 0.73 | 0.91 | -1.07 | 2.54 | 0.80 |
|  | **Autism vs ADHD × time** | **0.69** | **2.01** | **0.79** | **0.44** | **3.57** | **2.53*** |
| Sleep Anxiety | Diagnosis vs. no diagnosis | -0.30 | -0.49 | 0.27 | -1.02 | 0.03 | -1.86 |
|  | EL diagnosis vs. EL no diagnosis | -0.18 | -0.29 | 0.27 | -0.82 | 0.24 | -1.07 |
|  | Autism and ADHD vs. AuDHD | 0.03 | 0.05 | 0.55 | -1.03 | 1.14 | 0.10 |
|  | Autism vs ADHD | 0.08 | 0.14 | 0.43 | -0.70 | 0.97 | 0.32 |
|  | **Assessment time point** | **-0.20** | **-0.33** | **0.17** | **-0.66** | **-0.005** | **-2.01*** |
|  | sex | 0.04 | 0.06 | 0.21 | -0.36 | 0.48 | 0.28 |
|  | Diagnosis vs. no diagnosis × time | 0.10 | 0.16 | 0.31 | -0.44 | 0.77 | 0.54 |
|  | EL diagnosis vs. EL no diagnosis × time | 0.12 | 0.20 | 0.31 | -0.40 | 0.81 | 0.66 |
|  | Autism and ADHD vs. AuDHD × time | 0.56 | 0.91 | 0.60 | -0.28 | 2.11 | 1.51 |
|  | Autism vs ADHD × time | 0.49 | 0.80 | 0.52 | -0.22 | 1.83 | 1.56 |
| Night Wakings | Diagnosis vs. no diagnosis | -0.04 | -0.9 | 0.29 | -0.67 | 0.49 | -0.31 |
|  | EL diagnosis vs. EL no diagnosis | -0.09 | -0.16 | 0.30 | -0.75 | 0.42 | -0.55 |
|  | Autism and ADHD vs. AuDHD | -0.05 | -0.08 | 0.61 | -1.29 | 1.12 | -0.14 |
|  | Autism vs ADHD | 0.25 | 0.45 | 0.47 | -0.48 | 1.38 | 0.95 |
|  | Assessment time point | 0.03 | 0.06 | 0.19 | -0.32 | 0.44 | 0.32 |
|  | sex | 0.05 | 0.09 | 0.24 | -0.37 | 0.56 | 0.39 |
|  | Diagnosis vs. no diagnosis × time | 0.15 | 0.27 | 0.35 | -0.43 | 0.97 | 0.77 |
|  | EL diagnosis vs. EL no diagnosis × time | 0.003 | 0.01 | 0.35 | -0.69 | 0.71 | 0.02 |
|  | Autism and ADHD vs. AuDHD × time | 0.65 | 1.19 | 0.70 | -0.19 | 2.57 | 1.70 |
|  | Autism vs ADHD × time | 0.49 | 0.89 | 0.60 | -0.29 | 2.07 | 1.49 |
| Parasomnias | **Diagnosis vs. no diagnosis** | **-0.60** | **-1.04** | **0.28** | **-1.59** | **-0.49** | **-3.73***** |
|  | **EL diagnosis vs. EL no diagnosis** | **-0.46** | **-0.81** | **0.28** | **-1.36** | **-0.25** | **-2.85**** |
|  | Autism and ADHD vs. AuDHD | 0.45 | 0.79 | 0.59 | -0.36 | 1.93 | 1.36 |
|  | Autism vs ADHD | 0.46 | 0.81 | 0.45 | -0.08 | 1.70 | 1.80 |
|  | Assessment time point | 0.17 | 0.29 | 0.19 | -0.08 | 0.66 | 1.53 |
|  | **sex** | **0.29** | **0.51** | **0.23** | **0.07** | **0.95** | **2.27*** |
|  | Diagnosis vs. no diagnosis × time | 0.27 | 0.47 | 0.35 | -0.21 | 1.16 | 1.37 |
|  | EL diagnosis vs. EL no diagnosis × time | 0.31 | 0.54 | 0.35 | -0.15 | 1.23 | 1.55 |
|  | Autism and ADHD vs. AuDHD × time | -0.34 | -0.60 | 0.69 | -1.96 | 0.77 | -0.86 |
|  | Autism vs ADHD × time | 0.18 | 0.31 | 0.59 | -0.85 | 1.47 | 0.53 |
| Daytime sleepiness | Diagnosis vs. no diagnosis | -0.16 | -0.47 | 0.48 | -1.41 | 0.47 | -0.99 |
|  | EL diagnosis vs. EL no diagnosis | -0.14 | -0.43 | 0.48 | -1.38 | 0.52 | -0.89 |
|  | Autism and ADHD vs. AuDHD | 0.04 | 0.12 | 0.99 | -1.84 | 2.07 | 0.12 |
|  | Autism vs ADHD | -0.27 | -0.81 | 0.77 | -2.32 | 0.70 | -1.06 |
|  | **Assessment time point** | **-0.51** | **-1.51** | **0.32** | **-2.14** | **-0.88** | **-4.77***** |
|  | sex | 0.18 | 0.54 | 0.38 | -0.21 | 1.3 | 1.42 |
|  | Diagnosis vs. no diagnosis × time | -0.22 | -0.66 | 0.58 | -1.81 | 0.50 | -1.13 |
|  | EL diagnosis vs. EL no diagnosis × time | -012 | -0.35 | 0.59 | -1.51 | 0.81 | -0.60 |
|  | Autism and ADHD vs. AuDHD × time | -0.02 | -0.06 | 1.16 | -2.35 | 2.23 | -0.06 |
|  | Autism vs ADHD × time | 0.10 | 0.28 | 0.99 | -1.66 | 2.23 | 029 |

*Note.* EL = elevated likelihood; AuDHD = cooccurring autism and ADHD. *** p < .001. ** p < .01, * p < .05

**Table S4**

*Cumulative link mixed model predictors of sleep behaviors assessed with the Children’s Sleep Habits Questionnaire*

|  |  | OR | Log-odds | *SE* | *CI* | | z |
| --- | --- | --- | --- | --- | --- | --- | --- |
| DV | Predictor |  |  |  | *2.5%* | *97.5%* |  |
| Sleep onset delay | **Diagnosis vs. no diagnosis** | **0.44** | **-0.82** | **0.34** | **-1.50** | **-0.15** | **-2.40*** |
|  | EL diagnosis vs. EL no diagnosis | 0.53 | -0.63 | 0.34 | -1.29 | 0.04 | -1.84 |
|  | Autism and ADHD vs. AuDHD | 0.8 | -0.21 | 0.68 | -1.54 | 1.12 | -0.31 |
|  | Autism vs ADHD | 0.44 | -0.81 | 0.54 | -1.87 | 0.25 | -1.50 |
|  | Assessment time point | 1.08 | 0.07 | 0.3 | -0.51 | 0.66 | 0.25 |
|  | sex | 1.20 | 0.78 | 0.28 | -0.36 | 0.72 | 0.65 |
|  | Diagnosis vs. no diagnosis × time | 1.14 | 0.13 | 0.55 | -0.95 | 1.22 | 0.24 |
|  | EL diagnosis vs. EL no diagnosis × time | 1.54 | 0.43 | 0.56 | -0.66 | 1.52 | 0.78 |
|  | Autism and ADHD vs. AuDHD × time | 0.66 | -0.42 | 1.10 | -2.58 | 1.75 | -0.38 |
|  | Autism vs ADHD × time | 1.63 | 0.49 | 0.90 | -1.27 | 2.25 | 0.55 |
| Sleep duration score | **Diagnosis vs. no diagnosis** | **0.25** | **-1.40** | **0.52** | **-2.42** | **-0.39** | **-2.71**** |
|  | **EL diagnosis vs. EL no diagnosis** | **0.23** | **-1.46** | **0.53** | **-2.50** | **-0.42** | **-2.75**** |
|  | Autism and ADHD vs. AuDHD | 1.05 | 0.05 | 0.97 | -1.85 | 1.96 | 0.05 |
|  | Autism vs ADHD | 0.37 | -1.00 | 0.76 | -2.49 | 0.50 | -1.31 |
|  | Assessment time point | 0.85 | -0.16 | 0.35 | -0.85 | 0.53 | -0.46 |
|  | **sex** | **3.57** | **1.27** | **0.43** | **0.42** | **2.12** | **2.93**** |
|  | Diagnosis vs. no diagnosis × time | 1.39 | 0.33 | 0.65 | -0.95 | 1.61 | 0.51 |
|  | EL diagnosis vs. EL no diagnosis × time | 3.57 | 1.27 | 0.68 | -0.06 | 2.61 | 1.87 |
|  | Autism and ADHD vs. AuDHD × time | 0.61 | -0.50 | 1.30 | -3.05 | 2.04 | -0.39 |
|  | Autism vs ADHD × time | 0.85 | -0.16 | 1.06 | -2.24 | 1.92 | -0.15 |
| SDB | Diagnosis vs. no diagnosis | 0.42 | -0.87 | 0.52 | -1.89 | 0.15 | -1.68 |
|  | EL diagnosis vs. EL no diagnosis | 0.47 | -0.76 | 0.52 | -1.77 | 0.25 | -1.47 |
|  | Autism vs ADHD | 1.89 | 0.64 | 0.82 | -0.97 | 2.24 | 0.78 |
|  | Assessment time point | 1.16 | 0.15 | 0.40 | -0.64 | 0.94 | 0.38 |
|  | sex | 1.29 | 0.25 | 0.42 | -0.58 | 1.08 | 0.60 |
|  | Diagnosis vs. no diagnosis × time | 1.59 | 0.47 | 0.80 | -1.11 | 2.04 | 0.58 |
|  | EL diagnosis vs. EL no diagnosis × time | 1.71 | 0.54 | 0.80 | -1.03 | 2.10 | 0.67 |
|  | **Autism vs ADHD × time** | **0.04** | **-3.20** | **1.39** | **-5.93** | **-0.47** | **-2.30*** |

*Note.* EL = elevated likelihood; AuDHD = cooccurring autism and ADHD; SDB = sleep disordered breathing. *** p < .001. ** p < .01, * p < .05

**Table S5**

*Linear regression model predictors of changes in sleep behaviors from 10 to 14 months assessed with the Sleep and Settle Questionnaire*

|  |  | *Std. coeff* | *coeff* | *SE* | *CI* | | *t* |
| --- | --- | --- | --- | --- | --- | --- | --- |
| DV | Predictor |  |  |  | *2.5%* | *97.5%* |  |
| # of night awakenings | ADOS-2 sum CS | -0.01 | 0.01 | 0.09 | -0.19 | 0.17 | -0.10 |
|  | ADHD rating scale | -0.004 | -0.002 | 0.05 | -0.10 | 0.10 | -0.04 |
|  | Internalizing | -0.03 | -0.01 | 0.04 | -0.09 | 0.07 | -0.19 |
|  | externalizing | 0.25 | 0.05 | 0.03 | -0.01 | 0.10 | 1.61 |
|  | sex | 0.08 | 0.14 | 0.19 | -0.24 | 0.52 | 0.74 |
| # of night awakenings [EL] | ADOS-2 sum CS | 0.05 | 0.04 | 0.10 | -0.15 | 0.23 | 0.39 |
|  | ADHD rating scale | -0.09 | -0.04 | 0.05 | -0.14 | 0.07 | -0.72 |
|  | Internalizing | -0.10 | -0.02 | 0.04 | -0.11 | 0.06 | -0.61 |
|  | externalizing | 0.32 | 0.06 | 0.03 | 0.00 | 0.12 | 1.94 |
|  | sex | 0.06 | 0.11 | 0.21 | -0.31 | 0.52 | 0.51 |
| Settle duration | ADOS-2 sum CS | 0.05 | 0.29 | 0.63 | -7.17 | 2.84 | -0.86 |
|  | ADHD rating scale | 0.05 | 0.15 | 0.34 | -0.53 | 0.83 | 0.44 |
|  | Internalizing | 0.01 | 0.02 | 0.28 | -0.53 | 0.83 | 0.44 |
|  | externalizing | 0.07 | 0.09 | 0.20 | -0.31 | 0.48 | 0.43 |
|  | sex | -0.05 | -0.61 | 1.32 | -3.24 | 2.01 | -0.46 |
| Settle duration [EL] | ADOS-2 sum CS | 0.05 | 0.29 | 0.71 | -1.13 | 1.70 | 0.41 |
|  | ADHD rating scale | 0.04 | 0.12 | 0.39 | -0.65 | 0.89 | 0.30 |
|  | Internalizing | -0.02 | -0.04 | 0.31 | -0.66 | 0.58 | -0.13 |
|  | externalizing | 0.12 | 0.15 | 0.23 | -0.30 | 0.61 | 0.67 |
|  | sex | -0.04 | -0.55 | 1.55 | -3.63 | 2.54 | -0.35 |

*Note.* EL = elevated likelihood; ADOS-2 sum CS = ADOS-2 sum comparison score;

**Table S6**

*Linear regression model predictors of changes in sleep behaviors from 24 to 36 months assessed with the Children’s Sleep Habits Questionnaire*

|  |  | *Std. coeff* | *coeff* | *SE* | *CI* | | *t* |
| --- | --- | --- | --- | --- | --- | --- | --- |
| DV | Predictor |  |  |  | *2.5%* | *97.5%* |  |
| Sleep duration (hrs) | ADOS-2 sum CS | 0.09 | 0.04 | 0.06 | -0.08 | 0.17 | 0.72 |
|  | ADHD rating scale | -0.07 | -0.02 | 0.04 | -0.09 | 0.05 | -0.56 |
|  | Internalizing | 0.08 | 0.01 | 0.03 | -0.04 | 0.06 | 0.55 |
|  | externalizing | -0.04 | -0.01 | 0.02 | -0.04 | 0.03 | -0.26 |
|  | sex | 0.08 | 0.11 | 0.14 | -0.17 | 0.38 | 0.77 |
| Sleep duration (hrs) | ADOS-2 sum CS | 0.05 | 0.03 | 0.07 | -0.12 | 0.17 | 0.37 |
|  | ADHD rating scale | -0.02 | -0.01 | 0.04 | -0.09 | 0.07 | -0.18 |
|  | Internalizing | 0.12 | 0.02 | 0.03 | -0.04 | 0.08 | 0.70 |
|  | externalizing | -0.11 | -0.02 | 0.02 | -0.06 | 0.03 | -0.68 |
|  | sex | 0.05 | 0.06 | 0.16 | -0.26 | 0.38 | 0.37 |
| Total score | ADOS-2 sum CS | -0.19 | -0.47 | 0.27 | -1.01 | 0.07 | -1.71 |
|  | ADHD rating scale | -0.16 | -0.22 | 0.15 | -0.51 | 0.07 | -1.50 |
|  | Internalizing | -0.01 | -0.004 | 0.12 | -0.23 | 0.24 | 0.04 |
|  | externalizing | 0.10 | 0.07 | 0.09 | -0.12 | 0.25 | 0.71 |
|  | sex | 0.12 | 0.75 | 0.63 | -0.50 | 2.00 | 1.19 |
| Total score [EL] | ADOS-2 sum CS | -0.21 | -0.54 | 0.31 | -1.15 | 0.07 | -1.77 |
|  | ADHD rating scale | -0.16 | -0.22 | 0.16 | -0.55 | 0.10 | -1.37 |
|  | Internalizing | -0.04 | -0.03 | 0.13 | -0.30 | 0.24 | -0.23 |
|  | externalizing | 0.11 | 0.07 | 0.11 | -0.14 | 0.28 | 0.70 |
|  | sex | 0.06 | 0.40 | 0.73 | -1.05 | 1.86 | 0.55 |
| Bedtime resistance | ADOS-2 sum CS | -0.20 | -0.20 | 0.11 | -0.42 | 0.01 | -1.85 |
|  | ADHD rating scale | -0.07 | -0.04 | 0.06 | -0.16 | 0.08 | -0.68 |
|  | Internalizing | -0.17 | -0.06 | 0.05 | -0.15 | 0.04 | -1.17 |
|  | externalizing | 0.16 | 0.04 | 0.04 | -0.03 | 0.12 | 1.12 |
|  | sex | -0.02 | -0.06 | 0.25 | -0.56 | 0.44 | -0.24 |
| Bedtime resistance [EL] | **ADOS-2 sum CS** | **-0.25** | **-0.26** | **0.12** | **-0.49** | **-0.02** | **-2.14*** |
|  | ADHD rating scale | -0.08 | -0.04 | 0.06 | -0.17 | 0.08 | -0.69 |
|  | Internalizing | -0.20 | -0.07 | 0.05 | -0.17 | 0.04 | -1.25 |
|  | externalizing | 0.15 | 0.04 | 0.04 | -0.04 | 0.12 | 0.97 |
|  | sex | -0.03 | -0.08 | 0.29 | -0.65 | 0.49 | -0.28 |
| Sleep Anxiety | ADOS-2 sum CS | -0.14 | -0.09 | 0.07 | -0.22 | 0.05 | -1.23 |
|  | ADHD rating scale | -0.03 | -0.01 | 0.04 | -0.09 | 0.06 | -0.32 |
|  | Internalizing | 0.19 | 0.04 | 0.03 | -0.02 | 0.10 | 1.31 |
|  | externalizing | -0.08 | -0.01 | 0.02 | -0.06 | 0.03 | -0.56 |
|  | sex | 0.08 | 0.12 | 0.16 | -0.20 | 0.44 | 0.74 |
| Sleep Anxiety [EL] | ADOS-2 sum CS | -0.17 | -0.11 | 0.08 | -0.26 | 0.04 | -1.42 |
|  | ADHD rating scale | -0.001 | -0.002 | 0.04 | -0.09 | 0.08 | -0.06 |
|  | Internalizing | 0.19 | 0.04 | 0.03 | -0.03 | 0.11 | 1.20 |
|  | externalizing | -0.09 | -0.01 | 0.03 | -0.07 | 0.04 | -0.55 |
|  | sex | 0.07 | 0.11 | 0.19 | -0.26 | 0.48 | 0.58 |
| Night Wakings | ADOS-2 sum CS | -0.01 | 0.01 | 0.09 | -0.17 | 0.18 | 0.08 |
|  | ADHD rating scale | -0.14 | -0.06 | 0.05 | -0.15 | 0.03 | -1.26 |
|  | Internalizing | -0.06 | -0.02 | 0.04 | -0.09 | 0.06 | -044 |
|  | externalizing | -0.05 | -0.01 | 0.03 | -0.07 | 0.05 | -0.31 |
|  | sex | 0.04 | 0.08 | 0.20 | -0.31 | 0.48 | 0.42 |
| Night Wakings [EL] | ADOS-2 sum CS | 0.05 | 0.04 | 0.09 | -0.15 | 0.22 | 0.40 |
|  | ADHD rating scale | -0.10 | -0.04 | 0.05 | -0.14 | 0.06 | -0.84 |
|  | Internalizing | -0.04 | -0.01 | 0.04 | -0.09 | 0.07 | -0.24 |
|  | externalizing | -0.03 | -0.01 | 0.03 | -0.07 | 0.06 | -0.16 |
|  | sex | 0.06 | 0.11 | 0.22 | -0.33 | 0.54 | 0.49 |
| Parasomnias | **ADOS-2 sum CS** | **-0.29** | **-0.20** | **0.08** | **-0.35** | **-0.05** | **-2.61*** |
|  | ADHD rating scale | 0.10 | 0.04 | 0.04 | -0.04 | 0.12 | 0.90 |
|  | Internalizing | 0.10 | 0.02 | 0.03 | -0.04 | 0.09 | 0.68 |
|  | externalizing | 0.02 | 0.004 | 0.03 | -0.05 | 0.06 | 0.17 |
|  | sex | 0.18 | 0.31 | 0.17 | -0.03 | 0.65 | 1.79 |
| Parasomnias [EL] | **ADOS-2 sum CS** | **-0.26** | **-0.17** | **0.08** | **-0.33** | **-0.01** | **-2.16*** |
|  | ADHD rating scale | 0.05 | 0.02 | 0.04 | -0.07 | 0.11 | 0.47 |
|  | Internalizing | 0.09 | 0.02 | 0.04 | -0.05 | 0.09 | 0.59 |
|  | externalizing | 0.05 | 0.01 | 0.03 | -0.05 | 0.06 | 030 |
|  | sex | 0.18 | 0.31 | 0.19 | -0.07 | 0.69 | 1.61 |
| Daytime sleepiness | ADOS-2 sum CS | -0.02 | -0.02 | 0.13 | -0.29 | 0.25 | -0.14 |
|  | ADHD rating scale | -0.17 | -0.11 | 0.07 | -0.25 | 0.03 | -1.53 |
|  | Internalizing | -0.02 | -0.01 | 0.06 | -0.12 | 0.11 | -0.11 |
|  | externalizing | 0.12 | 0.04 | 0.05 | -0.05 | 0.13 | 0.81 |
|  | sex | 0.08 | 0.24 | 0.31 | -0.37 | 0.85 | 0.78 |
| Daytime sleepiness [EL] | ADOS-2 sum CS | -0.06 | -0.07 | 0.15 | -0.36 | 0.22 | -0.49 |
|  | ADHD rating scale | -0.16 | -0.10 | 0.08 | -0.26 | 0.05 | -1.31 |
|  | Internalizing | -0.08 | -0.03 | 0.06 | -0.16 | 0.10 | -0.46 |
|  | externalizing | 0.14 | 0.04 | 0.05 | -0.06 | 0.14 | 0.85 |
|  | sex | 0.03 | 0.10 | 0.35 | -0.60 | 0.80 | 0.29 |
| Sleep duration score | ADOS-2 sum CS | 0.02 | 0.01 | 0.06 | -0.10 | 0.12 | 0.14 |
|  | ADHD rating scale | -0.05 | -0.01 | 0.03 | -0.07 | 0.05 | -0.46 |
|  | Internalizing | 0.21 | 0.04 | 0.02 | -0.01 | 0.08 | 1.45 |
|  | externalizing | -0.09 | -0.01 | 0.02 | -0.05 | 0.03 | -0.59 |
|  | sex | 0.09 | 0.10 | 0.13 | -0.15 | 0.36 | 0.81 |
| Sleep duration score [EL] | ADOS-2 sum CS | 0.01 | 0.01 | 0.06 | -0.12 | 0.13 | 0.10 |
|  | ADHD rating scale | -0.09 | -0.03 | 0.03 | -0.09 | 0.04 | -0.77 |
|  | Internalizing | 0.15 | 0.02 | 0.03 | -0.03 | 0.08 | 0.91 |
|  | externalizing | -0.05 | -0.01 | 0.02 | -0.05 | 0.04 | -0.30 |
|  | sex | 0.01 | 0.01 | 0.15 | -0.28 | 0.31 | 0.09 |

*Note.* EL = elevated likelihood; ADOS-2 sum CS = Autism Diagnostic Observation Schedule -2 sum comparison score;

**Table S7**

*Cumulative link model predictors of changes in sleep behaviors from 24 to 36 months assessed with the Children’s Sleep Habits Questionnaire*

|  |  | OR | Log-odds | *SE* | *CI* | | z |
| --- | --- | --- | --- | --- | --- | --- | --- |
| DV | Predictor |  |  |  | *2.5%* | *97.5%* |  |
| Sleep onset delay | ADOS-2 sum CS | 0.90 | -0.11 | 0.09 | -0.27 | 0.06 | -1.25 |
|  | ADHD rating scale | 0.99 | -0.01 | 0.05 | -0.10 | 0.09 | -0.11 |
|  | Internalizing | 0.97 | -0.03 | 0.04 | -0.10 | 0.04 | -0.87 |
|  | externalizing | 1.02 | 0.02 | 0.03 | -0.04 | 0.08 | 0.64 |
|  | sex | 1.11 | 0.10 | 0.20 | -0.28 | 0.49 | 0.53 |
| Sleep onset delay [EL] | ADOS-2 sum CS | 0.90 | -0.10 | 0.09 | -0.29 | 0.08 | -1.13 |
|  | ADHD rating scale | 0.99 | -0.01 | 0.05 | -0.11 | 0.09 | -0.18 |
|  | Internalizing | 0.97 | -0.03 | 0.04 | -0.11 | 0.05 | -0.72 |
|  | externalizing | 1.01 | 0.01 | 0.03 | -0.05 | 0.08 | 0.31 |
|  | sex | 1.11 | 0.11 | 0.22 | -0.32 | 0.53 | 0.48 |
| SDB | ADOS-2 sum CS | 0.91 | -0.10 | 0.20 | -0.50 | 0.31 | -0.47 |
|  | ADHD rating scale | 0.85 | -0.16 | 0.10 | -0.36 | 0.04 | -1.59 |
|  | Internalizing | 1.25 | 0.22 | 0.13 | -0.03 | 0.47 | 1.74 |
|  | externalizing | 0.87 | -0.14 | 0.10 | -0.34 | 0.06 | -1.38 |
|  | sex | 0.32 | -1.14 | 0.58 | -2.29 | 0.00 | -1.96 |
| SDB [EL] | ADOS-2 sum CS | 0.70 | -0.36 | 0.44 | -1.22 | 0.50 | -0.82 |
|  | ADHD rating scale | 0.73 | 0.69 | 0.46 | -0.21 | 1.59 | 1.50 |
|  | internalizing | 1.99 | -0.61 | 0.42 | -1.44 | 0.22 | -1.44 |
|  | externalizing | 0.54 | -0.31 | 0.22 | -0.75 | 0.13 | -1.40 |
|  | sex | 0.01 | -4.49 | 2.98 | -10.32 | 1.34 | -1.51 |

*Note.* EL = elevated likelihood; ADOS-2 sum CS = Autism Diagnostic Observation Schedule-2 sum comparison score; SDB = sleep disordered breathing. *** p < .001. ** p < .01, * p < .05

**Table S8**

*Zero-order correlations of dimensional measures of autism and ADHD and externalizing and internalizing problems with sleep behaviors at 24 and 36 months assessed with the Children’s Sleep Habits Questionnaire*

| Sleep Behavior |  | Time assessment sleep behavior | r | p | p - adj |
| --- | --- | --- | --- | --- | --- |
| Bedtime resistance | ADHD rating scale | 24 months | 0.08 | 0.376 | 1 |
| Bedtime resistance | ADHD rating scale | 36 months | 0.01 | 0.92 | 1 |
| Bedtime resistance | ADOS-2 sum CS | 24 months | 0.10 | 0.238 | 1 |
| Bedtime resistance | ADOS-2 sum CS | 36 months | -0.07 | 0.339 | 1 |
| Bedtime resistance | externalizing | 24 months | 0.18 | 0.0297 | 1 |
| Bedtime resistance | externalizing | 36 months | 0.17 | 0.021 | 1 |
| Bedtime resistance | internalizing | 24 months | 0.21 | 0.0105 | 0.578 |
| Bedtime resistance | internalizing | 36 months | 0.15 | 0.0449 | 1 |
| Daytime Sleepiness | ADHD rating scale | 24 months | 0.01 | 0.915 | 1 |
| Daytime Sleepiness | ADHD rating scale | 36 months | -0.11 | 0.206 | 1 |
| Daytime Sleepiness | ADOS-2 sum CS | 24 months | 0.08 | 0.347 | 1 |
| Daytime Sleepiness | ADOS-2 sum CS | 36 months | 0.06 | 0.412 | 1 |
| Daytime Sleepiness | externalizing | 24 months | 0.08 | 0.351 | 1 |
| Daytime Sleepiness | externalizing | 36 months | 0.15 | 0.0427 | 1 |
| Daytime Sleepiness | internalizing | 24 months | 0.22 | 0.00747 | 0.433 |
| Daytime Sleepiness | internalizing | 36 months | 0.19 | 0.00894 | 0.501 |
| Night wakings | ADHD rating scale | 24 months | 0.03 | 0.728 | 1 |
| Night wakings | ADHD rating scale | 36 months | -0.03 | 0.739 | 1 |
| Night wakings | ADOS-2 sum CS | 24 months | 0.00 | 0.954 | 1 |
| Night wakings | ADOS-2 sum CS | 36 months | -0.03 | 0.711 | 1 |
| Night wakings | externalizing | 24 months | 0.16 | 0.0604 | 1 |
| Night wakings | externalizing | 36 months | 0.08 | 0.302 | 1 |
| Night wakings | internalizing | 24 months | 0.18 | 0.0342 | 1 |
| Night wakings | internalizing | 36 months | 0.05 | 0.533 | 1 |
| Parasomnias | ADHD rating scale | 24 months | 0.02 | 0.809 | 1 |
| Parasomnias | ADHD rating scale | 36 months | 0.13 | 0.118 | 1 |
| Parasomnias | ADOS-2 sum CS | 24 months | 0.17 | 0.0385 | 1 |
| Parasomnias | ADOS-2 sum CS | 36 months | 0.00 | 1 | 1 |
| Parasomnias | externalizing | 24 months | 0.31 | 0.0002 | **0.012** |
| Parasomnias | externalizing | 36 months | 0.30 | 3.4E-05 | **0.003** |
| Parasomnias | internalizing | 24 months | 0.29 | 0.0003 | **0.023** |
| Parasomnias | internalizing | 36 months | 0.23 | 0.002 | 0.115 |
| SDB | ADHD rating scale | 24 months | 0.15 | 0.109 | 1 |
| SDB | ADHD rating scale | 36 months | 0.02 | 0.8 | 1 |
| SDB | ADOS-2 sum CS | 24 months | 0.04 | 0.675 | 1 |
| SDB | ADOS-2 sum CS | 36 months | 0.06 | 0.431 | 1 |
| SDB | externalizing | 24 months | 0.19 | 0.021 | 1 |
| SDB | externalizing | 36 months | 0.23 | 0.001 | 0.091 |
| SDB | internalizing | 24 months | 0.20 | 0.019 | 0.981 |
| SDB | internalizing | 36 months | 0.20 | 0.008 | 0.444 |
| sleep duration (hrs) | ADHD rating scale | 24 months | 0.02 | 0.796 | 1 |
| sleep duration (hrs) | ADHD rating scale | 36 months | -0.08 | 0.341 | 1 |
| sleep duration (hrs) | ADOS-2 sum CS | 24 months | -0.07 | 0.408 | 1 |
| sleep duration (hrs) | ADOS-2 sum CS | 36 months | -0.06 | 0.415 | 1 |
| sleep duration (hrs) | externalizing | 24 months | -0.07 | 0.419 | 1 |
| sleep duration (hrs) | externalizing | 36 months | -0.13 | 0.085 | 1 |
| sleep duration (hrs) | internalizing | 24 months | -0.10 | 0.254 | 1 |
| sleep duration (hrs) | internalizing | 36 months | -0.18 | 0.016 | 0.875 |
| Sleep anxiety | ADHD rating scale | 24 months | 0.04 | 0.696 | 1 |
| Sleep anxiety | ADHD rating scale | 36 months | 0.00 | 0.979 | 1 |
| Sleep anxiety | ADOS-2 sum CS | 24 months | 0.04 | 0.598 | 1 |
| Sleep anxiety | ADOS-2 sum CS | 36 months | -0.08 | 0.261 | 1 |
| Sleep anxiety | externalizing | 24 months | 0.28 | 0.001 | **0.046** |
| Sleep anxiety | externalizing | 36 months | 0.20 | 0.006 | 0.372 |
| Sleep anxiety | internalizing | 24 months | 0.28 | 0.001 | **0.044** |
| Sleep anxiety | internalizing | 36 months | 0.21 | 0.004 | 0.260 |
| Sleep duration score | ADHD rating scale | 24 months | 0.09 | 0.322 | 1 |
| Sleep duration score | ADHD rating scale | 36 months | 0.08 | 0.354 | 1 |
| Sleep duration score | ADOS-2 sum CS | 24 months | 0.22 | 0.006 | 0.364 |
| Sleep duration score | ADOS-2 sum CS | 36 months | 0.14 | 0.055 | 1 |
| Sleep duration score | externalizing | 24 months | 0.30 | 0.0002 | **0.014** |
| Sleep duration score | externalizing | 36 months | 0.30 | 3.6E-05 | **0.003** |
| Sleep duration score | internalizing | 24 months | 0.36 | 8.9E-06 | **0.001** |
| Sleep duration score | internalizing | 36 months | 0.34 | 1.6E-06 | **0.0001** |
| Sleep onset delay | ADHD rating scale | 24 months | 0.12 | 0.207 | 1 |
| Sleep onset delay | ADHD rating scale | 36 months | 0.11 | 0.193 | 1 |
| Sleep onset delay | ADOS-2 sum CS | 24 months | 0.14 | 0.092 | 1 |
| Sleep onset delay | ADOS-2 sum CS | 36 months | 0.02 | 0.753 | 1 |
| Sleep onset delay | externalizing | 24 months | 0.24 | 0.004 | 0.235 |
| Sleep onset delay | externalizing | 36 months | 0.25 | 0.0007 | **0.046** |
| Sleep onset delay | internalizing | 24 months | 0.29 | 0.0004 | **0.025** |
| Sleep onset delay | internalizing | 36 months | 0.26 | 0.0004 | **0.025** |
| total score | ADHD rating scale | 24 months | 0.09 | 0.313 | 1 |
| total score | ADHD rating scale | 36 months | 0.02 | 0.807 | 1 |
| total score | ADOS-2 sum CS | 24 months | 0.16 | 0.048 | 1 |
| total score | ADOS-2 sum CS | 36 months | 0.03 | 0.66 | 1 |
| total score | externalizing | 24 months | 0.33 | 4.4E-05 | **0.003** |
| total score | externalizing | 36 months | 0.35 | 1.5E-06 | **0.0001** |
| total score | internalizing | 24 months | 0.41 | 4E-07 | **3.2E-05** |
| total score | internalizing | 36 months | 0.34 | 2.6E-06 | **0.0002** |

*Note.* ADOS-2 sum CS = ADOS-2 sum comparison score; SDB = sleep disordered breathing, p-adj: adjusted using holm method.
